# Regulation of Zebrafish Hatching by Tetraspanin *cd63*

**DOI:** 10.1371/journal.pone.0019683

**Published:** 2011-05-19

**Authors:** Michael Z. Trikić, Pete Monk, Henry Roehl, Lynda J. Partridge

**Affiliations:** 1 Molecular Biology and Biotechnology, University of Sheffield, Sheffield, United Kingdom; 2 Department of Infection and Immunity, University of Sheffield, Sheffield, United Kingdom; 3 Department of Biomedical Science, University of Sheffield, Sheffield, United Kingdom; University Paris Sud, France

## Abstract

Tetraspanins cause the clustering of membrane proteins into a level of organisation essential for cellular function. Given the importance and complicated nature of this mechanism, we attempted a novel approach to identify the function of a single component in a biologically relevant context. A morpholino knockdown strategy was used to investigate the role of *cd63*, a membrane protein associated with intracellular transport and a melanoma marker, in embryonic zebrafish. By using three separate morpholinos targeting *cd63*, we were able to identify a specific phenotype. Strikingly, morphant fish failed to hatch due to the lack of secreted proteolytic enzymes required for chorion-softening. The morphology of the hatching gland at both the cellular and intracellular levels was disorganised, suggesting a role for *cd63* in the functioning of this organ. This work identifies a specific role for *cd63* in the zebrafish embryo and provides evidence for the suitability of zebrafish as a model system for the investigation of tetraspanin enriched microdomains.

## Introduction

CD63 is a member of the tetraspanin superfamily of four-span membrane proteins, distinguished by the presence of 4–8 cysteine residues in the second extracellular domain (EC2) in specific sequence motifs and conserved polar residues in the transmembrane domains [1]. Tetraspanins have the ability to cause the clustering of membrane proteins in tetraspanin-enriched microdomains (TEMs), a level of organisation essential for cellular function [2]. Human CD63 was first reported as the ME491 antigen, a tumour marker [3] and is now more commonly known as a marker of lysosomes and multivesicular bodies. The movement of CD63 from intracellular organelles to the cell surface during secretion has led to its use as an activation marker for several haematopoietic cell types including neutrophils, eosinophils, basophils, mast cells and platelets [4]. It is also present in melanosomes, cytotoxic T cell granules, Weibel-Palade bodies of endothelial cells, MHCII compartments of dendritic cells [5] and is enriched on exosomes [6]. All mammalian cell types so far studied express CD63, suggesting that it may be ubiquitous [5]. Many proteins are known to associate directly with CD63, including H^+^/K^+^ ATPase β subunit [7], syntenin 1 [8], MT1-MMP1 [9], PI-4-kinase [10], AP3 [11], TIMP1 [12], amelogenin [13] and PTK [14]. In many cases, the association with CD63 relates to the cellular trafficking of the partner protein: MT1-MMP1 is targeted by CD63 for lysosomal degradation, as is the β subunit of H^+^/K^+^ ATPase and amelogenin. CXCR4 surface expression [15] and the delivery of elastase to neutrophil primary granules (a form of secretory lysosome) [16] appears to be controlled by CD63. In rat basophilic leukaemia mast cells (RBL-2H3) CD63 is expressed in secretory lysosomes [17], were it is critical for full degranulation [18]. The protease cathepsin L (CatL), which has both intracellular roles (i.e. neuropeptide processing in chromaffin cells [19]; transcription factor activation in the nucleus [20], [21]) and extracellular roles (i.e. cancer cell migration [22] and extracellular matrix degradation [23]), also co-localizes with CD63, although it is not known if these molecules are co-trafficked [24]. Phylogenetic analysis has described the CD63 family as constituting one of the four major families of vertebrate tetraspanins [25]. CD63 is likely to have had a particularly ancient origin, as it is associated with a gene expansion in Drosophila and is also reported in sponges [26]. The zebrafish homologue is highly expressed from an early developmental stage in the pre-polster [27], a tissue which gives rise to the hatching gland, and has a key role in patterning of the embryo [28].

Although members of the tetraspanin superfamily are well represented in the zebrafish genome, little work has been conducted on their role in fish development. For this reason we chose to investigate CD63 in embryonic zebrafish. Expression during early development was confirmed and the role of CD63 investigated using antisense morpholino-mediated knockdown. Strikingly, morphant fish failed to hatch, due to the lack of secreted proteolytic enzymes required for chorion-softening. The morphology of the hatching gland at both the cellular and intracellular levels was disorganised, suggesting a role for CD63 in the function of this organ.

## Results

### Analysis of CD63 protein sequence

**Figure 1:**
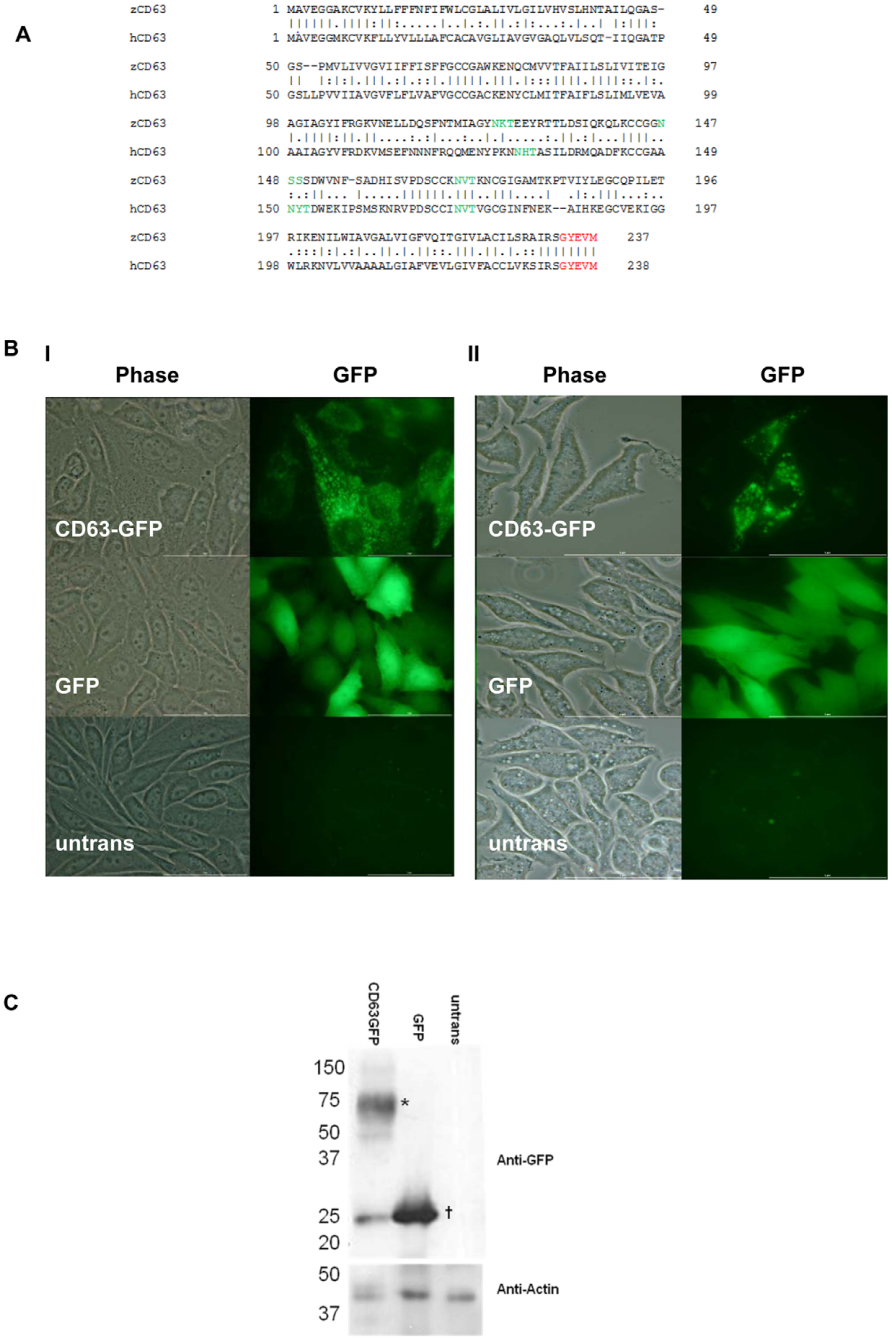
CD63 protein properties. A. Alignment between *Homo sapiens* (CAG46893) and *Danio rerio* (NP_955837). Functional motifs are highlighted; glycosylation sites are in green, lysosomal targeting motif in red. BI. CHO (Chinese Hamster ovary) cells, and BII. RBL-2H3 (rat basophilic leaukaemia) cells, transfected with Cd63-GFP, GFP, or untransfected. C. Western blot of lysates from CHO cells probed with anti GFP, with anti actin as a loading control. Ladder positions are indicated on the left in kDa. * denotes Cd63GFP, **†** denotes GFP.

The zebrafish Cd63 molecule is 45.4% identical (62.2% similar) to human CD63 and the key structural features, such as the three glycosylation sites in the large extracellular domain and the internalization motif at the C-terminus, are retained (Figures 1A and S1). GFP-tagged zebrafish Cd63 expressed in mammalian cell lines (CHO and RBL-2H3) and live embryos, displayed the intracellular localisation typical of CD63 in other species (Figures 1BI and II and S2A and B) [29]. Western blot analysis of lysates using anti-GFP antibodies revealed a smeared banding pattern from ∼50–75 kDa in Cd63GFP transfected CHO cells, indicating heterogeneous glycosylation of Cd63 as reported for the human protein [30], [31], [32] (Figure 1C). Similar results were seen with RBL-2H3 and fish lysates.

### Investigation of *cd63* in embryonic zebrafish

**Figure 2:**
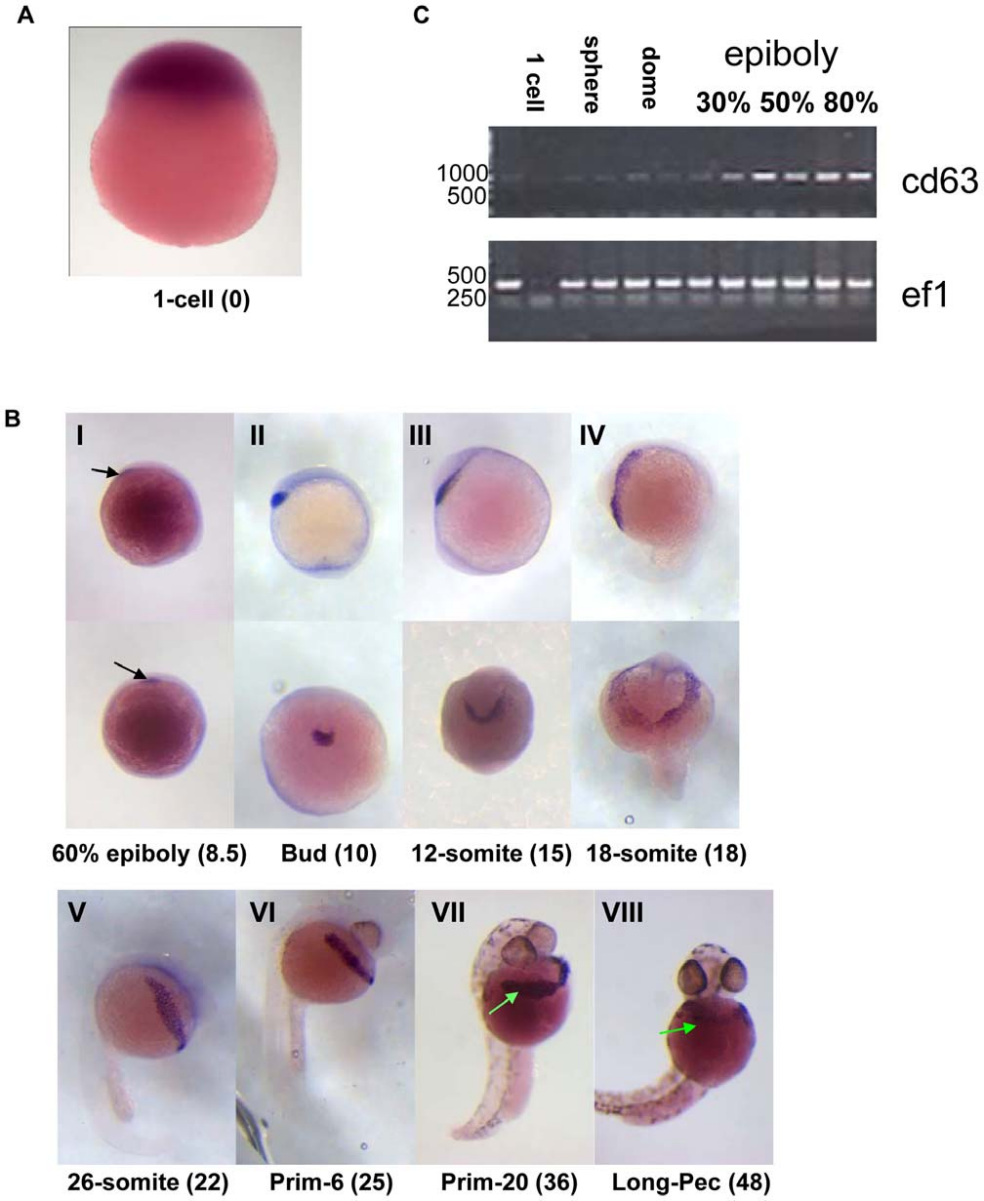
*cd63* transcript. A & B. Location of *cd63* transcript as revealed by ISH, using a probe against *cd63*. Developmental stages are noted below with hpf (hours post fertilisation) in brackets. In the top panel of B (I–IV), the top row gives the lateral view, bottom row ventral. In BI, arrows indicate the first expression in the pre-polster. *cd63* expression continues (BII–VII). Expression in the hatching gland is indicated in BVII and BVIII, by arrows. C. RT-PCR time course using single embryos. PCR primers are indicated on the right, *ef1* is a ubiquitous transcription factor used as a control. Numbers indicate ladder position in base pairs. Developmental stages are indicated at the top of the figure, each carried out in duplicate. This gel is representative of three replicates.

Existing ISH data indicates that zygotic *cd63* transcripts are expressed during early development [27], [33]. Here, we have confirmed and extended these observations. Maternally-derived *cd63* transcript (pre-zygotic transcription) is present in the early stages of embryonic development with positive staining for *cd63* at the 1 cell stage (Figure 2A) and *cd63* positive RT-PCR bands from the 1-cell to dome stage embryos (Figure 2C). Using ISH, zygotic *cd63* expression was first detectable between 6–8 hpf in the pre-polster, located under the forebrain (Figure 2B I, black arrow; Figure 2C 30% epiboly). This structure gives rise to the polster where *cd63* expression continues and increases. The polster is the precursor to the hatching gland, which develops and spreads (Figure 2B III-IV), with cells finally migrating across the yolk (Figure 2B V–VII) to form a mature hatching gland (Figure 2B VII, arrow). After hatching (a heterogeneous event within a clutch occurring between 48 and 72 hours post fertilisation [34], [35]), *cd63* levels in the hatching gland diminish (Figure 2B VIII, arrow).

### Knockdown of *cd63*

**Figure 3:**
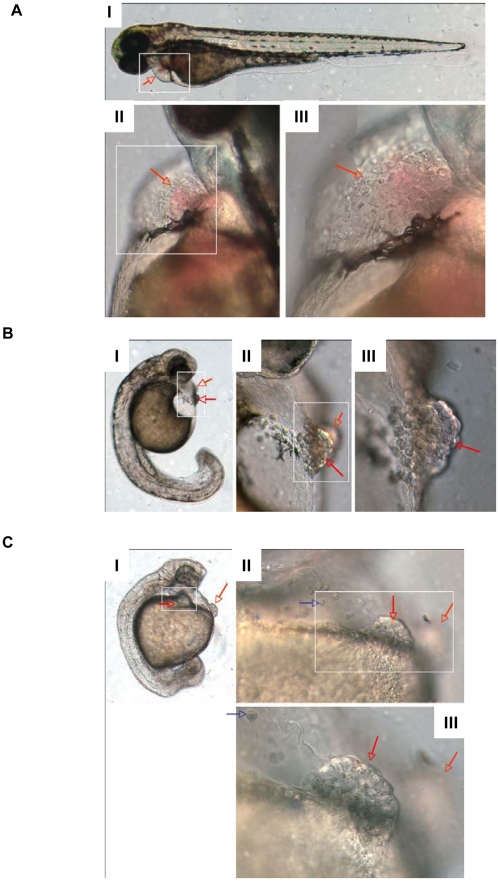
Morphology of *cd63* knockdown. DIC microscopy images of the hatching glands of dechorinated 52 hour post fertilisation embryos. In each panel (A–C) I is x5 objective, white box indicates frame of II. II is x20 objective, white box indicates frame of III. III is x40 objective. II and III are montages of more than one focal plane. Red arrows point to the hatching gland, blue arrows indicate mislocalised hatching gland cells. A: Hatching gland typical of a LWT embryo. I is a montage of more than one focal plane. B: Moderately disrupted hatching gland typical after injection of 10 pg of MO4. C: Severely disrupted hatching gland typical after injection of 12 pg of MO4.

**Figure 4:**
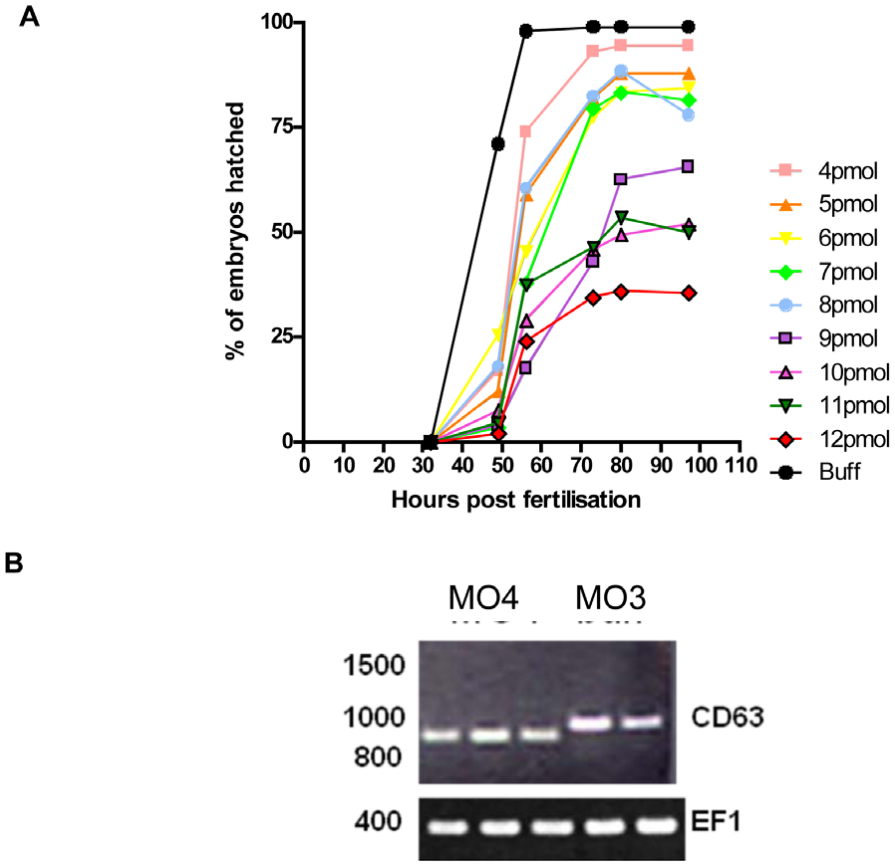
Effect of knockdown. A. Effect of morpholino-induced knockdown of *cd63* on hatching. Buff =  buffer/mismatch injected control. Units of time are hours. Data from two experiments each using MO2 and 4 and a combined total of 671 embryos. B. MO4 mediated band shift. 1% Agarose gel of RT-PCR products from 48 hr LWT embryos using primers corresponding to full length *cd63* transcript or *ef1*, a ubiquitous transcription factor used as a control. The 3 lanes next to the ladder are from embryos injected with 10 pg MO4, remaining lanes are from mismatch MO3 injected embryos. Similar results were seen in the 30 MO4 injected embryos tested.

To investigate the role of *cd63* in zebrafish development, we used morpholinos to knockdown gene function. *Danio* embryos were injected with morpholinos targeting the translation start site (MO1) and two intron/exon boundaries (MO2, 4) along with a MO1 mismatch control (MO3), to observe the effects of *cd63* knockdown on *Danio* development. MO1, MO2, MO4 but not MO3 caused a failure of apparently viable embryos to hatch, even when fish embryos appeared grossly normal (Figure 3BI and CI), despite MO induced non specific defects [36]. To investigate the hatching defect seen in morphants, a titration of MO against hatching was carried out to confirm that the phenotype was dose dependent. Embryos were allowed to develop until 100 hours post fertilisation at which point the % of hatched embryos was calculated. A dose-dependent inhibition of hatching was seen in morphant embryos, manifested as failure to hatch or a delay in hatching compared to control embryos (Figure 4A), suggesting that *cd63* has a specific role in the mechanism of hatching.

RT-PCR investigation of transcripts from embryos injected with splice blocking MO4 provided further evidence of specific targeting: PCR products generated from mis-spliced transcript revealed a truncation of the 75 base pairs of exon 4 at the point of MO4 targeting, confirmed by sequencing of embryo derived transcript and visible on agarose gels (Figure 4B).

### Relation of *cd63* to the Nodal signalling pathway and hatching gland specification

**Figure 5:**
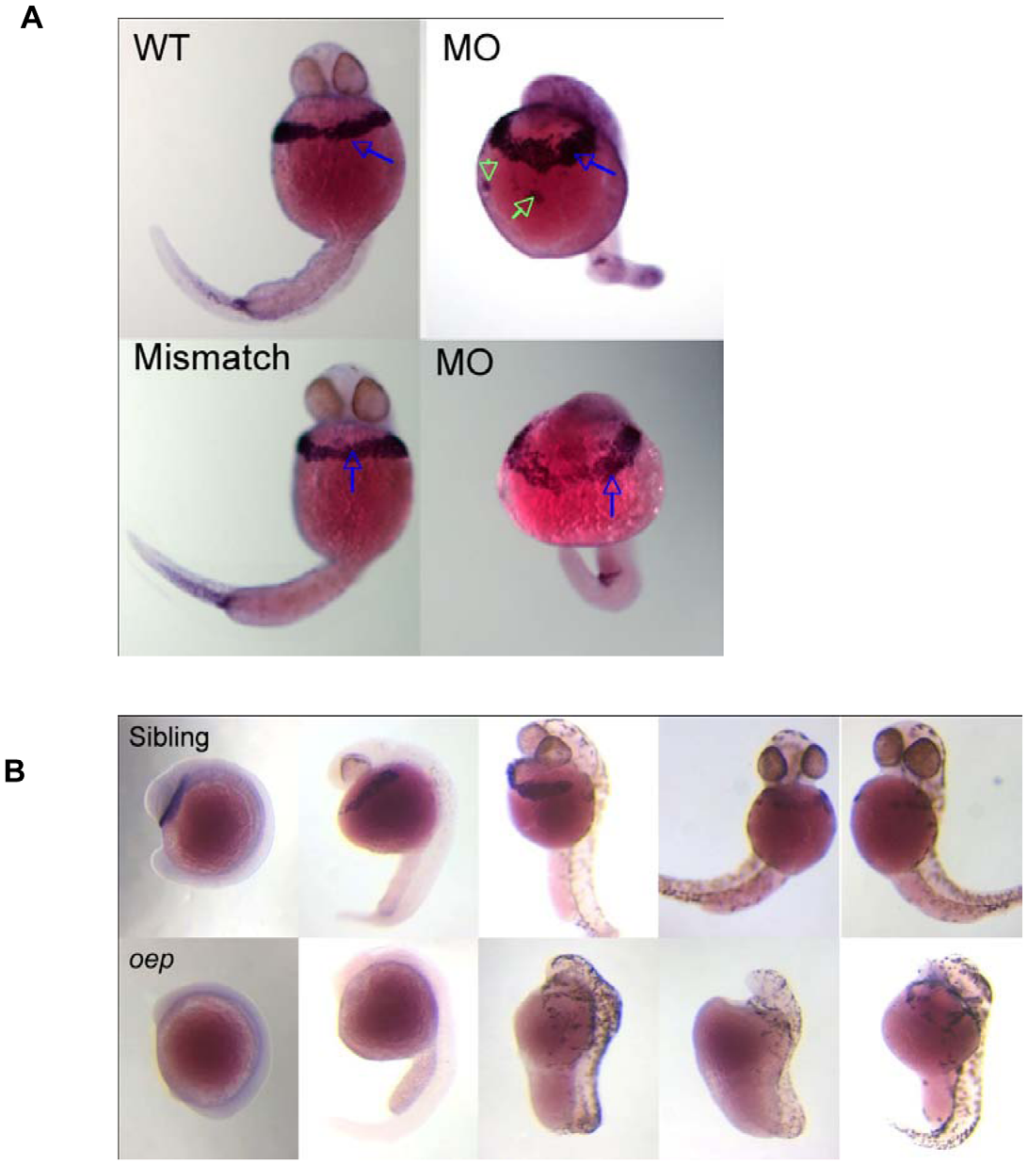
Location of *cd63* transcript as revealed by ISH. A. 32 h.p.f LWT dechorinated embryos injected with 10 pg MO4 as indicated. Blue arrows indicates hatching gland; green arrows denote mis-localised hatching gland cell clumps B. One Eyed Pinhead (*oep*) mutants and siblings. The top row are sibling embryos homozygous or heterozygous for WT *oep*, where as the bottom row are homozygous for the *oep* deletion.

Given the observed hatching defect as a result of *cd63* knockdown, we attempted to determine if the influence of *cd63* on hatching gland formation and function was due to a position in a signalling pathway responsible for hatching gland specification. Firstly, we tested *cd63* expression in morphants using ISH (Figure 5A). This demonstrated that a consequence of knockdown is organisational disruption to hatching gland tissue in the absence of changes in tissue density (Figure 5A). Therefore it would appear that *cd63* knockdown does not result in a reduction in hatching gland tissue and so *cd63* is not required for specification of the pre-hatching gland cells fated to become hatching gland cells.

**Figure 6:**
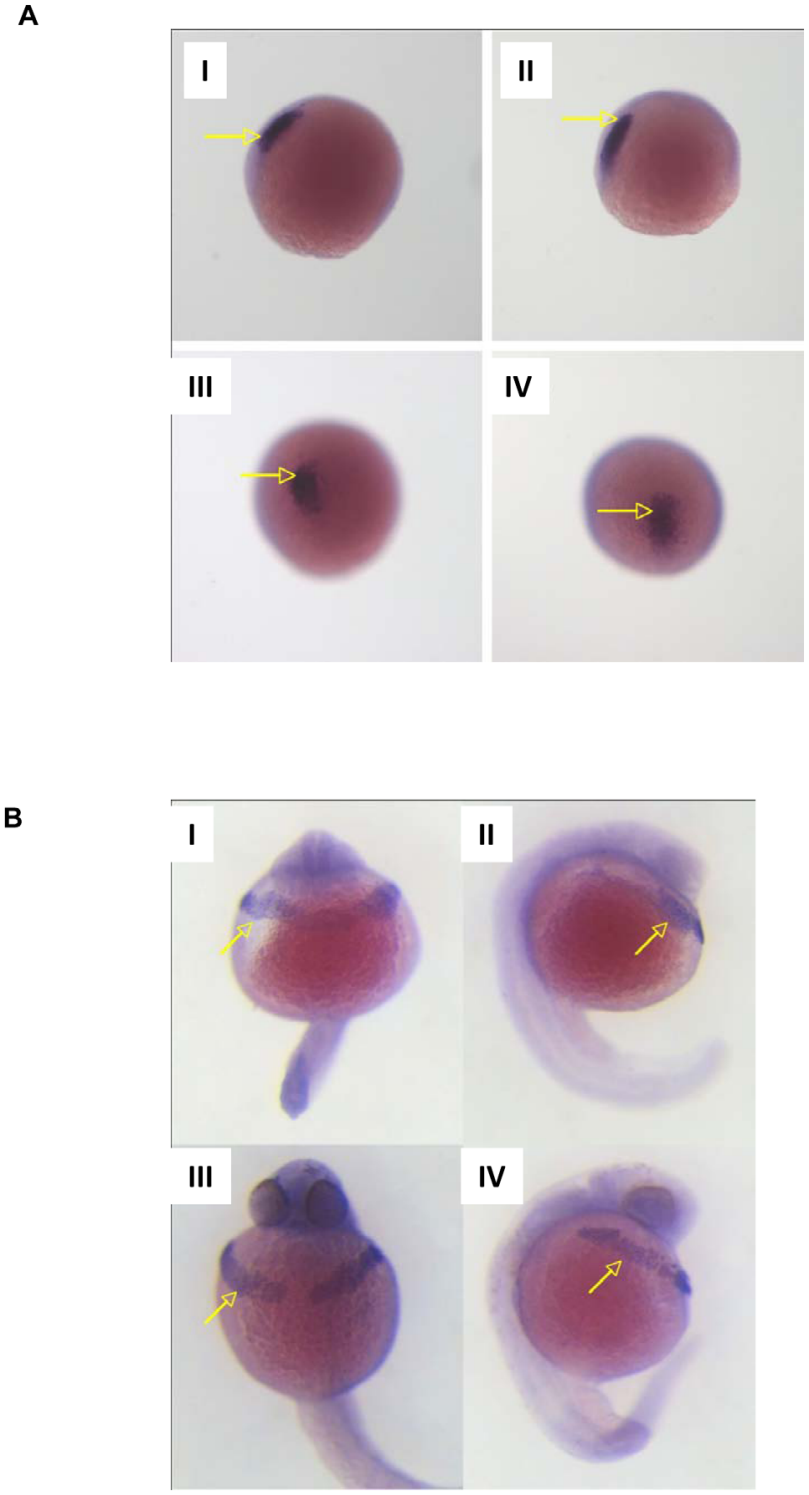
Determination of mesodermal markers by ISH. Morphant embryos were injected with 10 pg MO4. A. Location of pre-chordal plate marker *gsc* in LWT embryos at 80% epiboly. I and II give lateral view, III and IV looking down on *gsc* positive staining. II and IV were injected with MO4, I and III are mis-match injected siblings. Arrows indicate *gsc* specific staining. B. Hatching gland marker *cat L* in 24 hour post fertilisation LWT embryos. I and II are morphant embryos, ventral and lateral views respectively. III and IV are mismatch injected LWT embryos, ventral and lateral views respectively. Arrows indicate hatching gland specific *cat L* staining.

*cd63* is highly expressed from an early developmental stage in structures derived from the pre-chordal plate, formation of which relies on the Nodal signalling pathway. To address the possibility that *cd63* might be involved in the Nodal signalling pathway, the Nodal deficient mutant *oep* was probed for expression of *cd63*. *Oep* mutants have a complete absence of *cd63* staining (Figure 5B, bottom row), whereas WT siblings have strong hatching gland staining as well as notochord staining (Figure 5B, top row). This demonstrates that *cd63* is Nodally regulated and therefore any role of *cd63* in tissue differentiation occurs downstream of Nodal signalling. To test for defects in dorsal mesoderm specification as a consequence of *cd63* knockdown, ISH was used to probe for the marker of dorsal mesoderm *gsc.* A change in *gsc* expression in the prechordal plate caused by *cd63* knockdown could have downstream influence on tissues derived from here, including the hatching gland. No difference was seen in *gsc* expression patterns between morphant embryos and uninjected controls in the pre-chordal plate (Figure 6A, arrows), suggesting that *cd63* operates downstream of *gsc.* To further explore a potential role for *cd63* in the hatching gland expression of hatching gland marker *cathepsin L* (*cat L*) [37] was investigated in morphants using ISH. Morphant embryos showed no difference in expression levels of *cat L* compared to WT controls (Figure 6B, arrows). This indicates that *cd63* is not involved in formation of the hatching gland tissue, as *cat L* is present at equivalent levels in the morphant and control. *cd63* knock down does not prevent formation of the hatching gland or cause a reduction in hatching gland tissue density. Taken together this suggests that *cd63* is not involved in specification of hatching gland tissues from precursor cells, but may be important for terminal differentiation of the secretory machinery and is required for the proper hatching gland function.

### Hatching gland phenotype alteration due to knockdown

**Figure 7:**
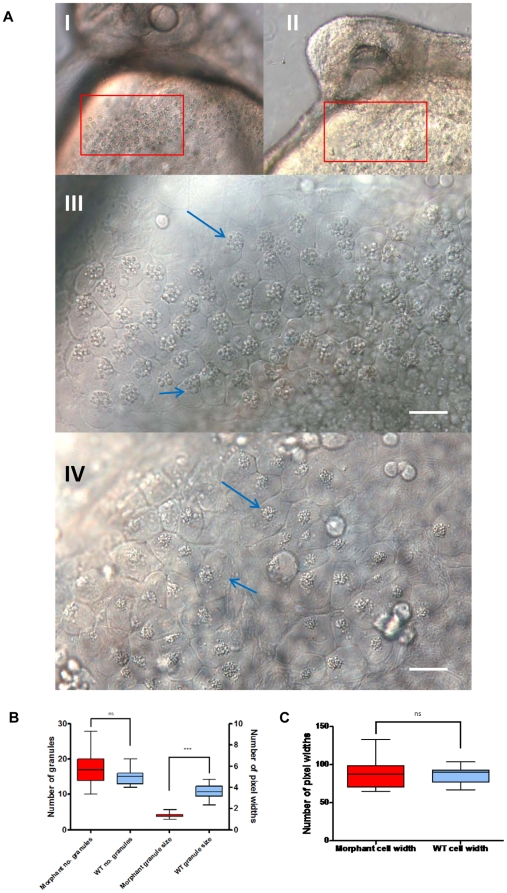
Morphant hatching gland. A. DIC microscopy images of cells of the hatching gland in a dechorinated control embryo (I and III) and morphant (II and IV) at 32 hours post fertilisation. III and IV are areas corresponding to the area of the red box in I and II respectively. Blue arrows denote intracellular granules. B and C. Analysis of hatching gland cell characteristics. Images of 3 wild type hatching glands and 3 morphant hatching glands were used for analysis with ImageJ. Distribution of data was tested to determine the relevant statistical analysis for each parameter measured. B: From each image five cells were randomly selected. Granule number was calculated and five granules randomly selected and measured. 2 tailed t-test of morphant vs. wt no. of granules per cell- no significant difference; Wilcoxon matched pairs test of morphant vs. wt granule size− p = <0.0001 *** significantly different. C: From each image five cells were randomly selected and measured at the widest diameter. 2 tailed t-test of morphant vs. wt cell size- no significant difference.

On closer inspection of embryos, morpholino-mediated *cd63* knockdown disrupted the organisation of the hatching gland. In contrast to the typical band of hatching gland cells, which narrow centrally and span the yolk surface under the face in WT and mismatch embryos (red arrows Figure 3A and Figure 7A I, and blue arrows Figure 5A WT and Mismatch), embryos injected with MO4 or MO1, MO2 (data not shown) underwent aberrant migration (Figure 3C, blue arrows; Figure 5A, MO, blue arrows; Figure 7II) with hatching gland cells often aggregating into two balls located each side of the yolk centre (Red arrows Figure 3B I–III and 3C I–III; green arrows Figure 5A). A striking characteristic of cells of the hatching gland are the large, bulbous intracellular granules which are normally seen tightly clustered together in an approximately central location (Figure 7A I and III, blue arrows).

### Granule motility changes as a result of *cd63* knockdown

The second defect seen in morphants was transformation of the granules, which were significantly smaller and showed loose clustering (Figures 7A IV, blue arrows and Figure 7B). Further to this, use of a x40 objective revealed barely perceptible granule movement in control cells (Movie S1), whilst the granules in morphant cells rapidly moved around in a seemingly chaotic manner (Movie S2). Although granule morphology was altered, no difference in granule number per cell or hatching gland cell size was seen (Figure 7 B and C). Attempted RNA-mediated rescue of this phenotype was unsuccessful, presumably due to over expression phenotypes seen as a result of *cd63* RNA injection, and limited stability of RNA (data not shown).

### The failure to hatch is due to a loss of hatching enzyme release

**Figure 8:**
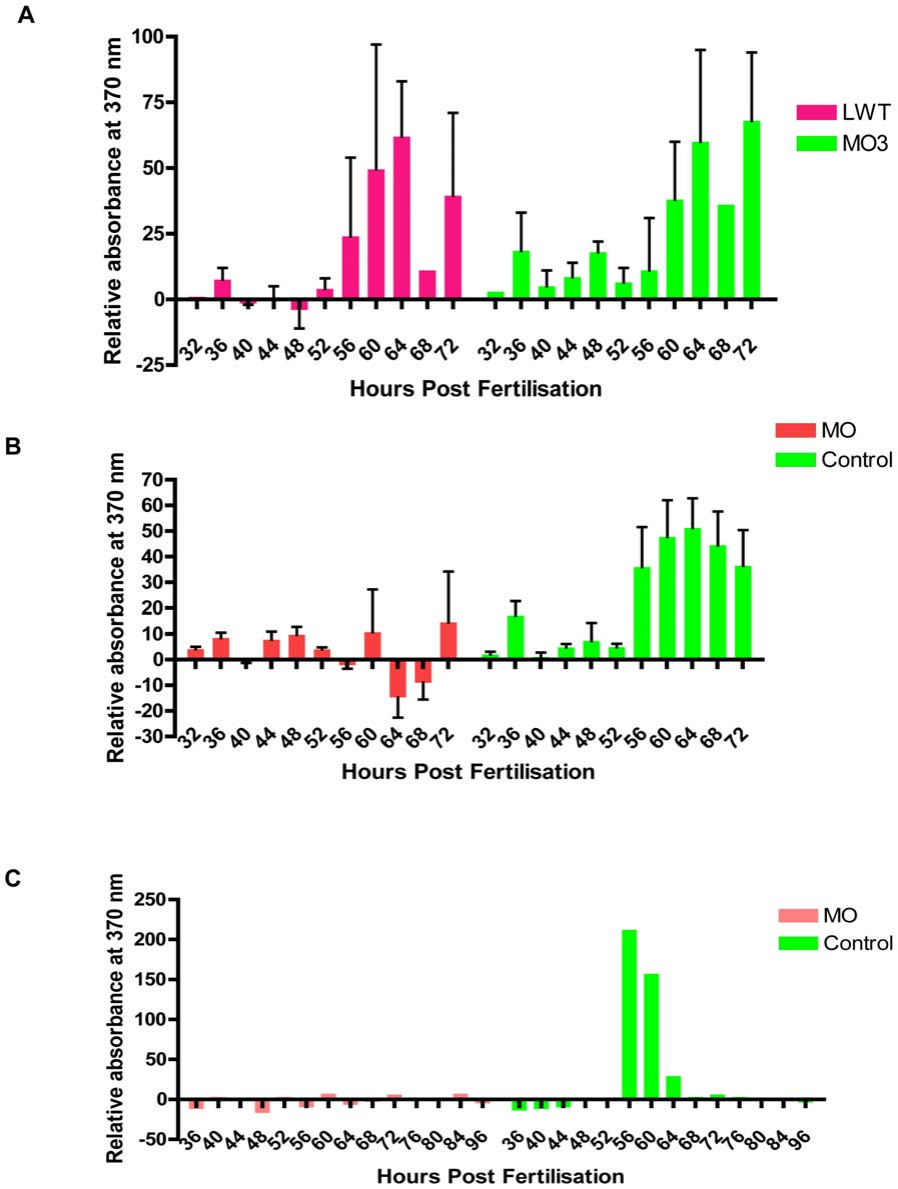
Assay for hatching enzymes. Media from the wells containing each embryo was harvested on a four hourly basis and tested for substrate cleavage ability determined by measuring release of fluorescent substrate. Doses of 10 pg of MO2 or 4 were injected, 0.8 pg MO1. A. Single control embryos. LWT are uninjected embryos. 2 way ANOVA of effect of MO3(mismatch) vs. uninjected embryos- % total variation 1.35, p = <0.46 ns. n = 2; total of 27 embryos. B. Assay for hatching enzymes in single MO1, MO2 and MO4 injected embryos. Control embryos were uninjected or MO3 injected. 2 way ANOVA of effect of MO vs. Control- % total variation 5.68, p = <0.0001 *** significant. n = 2; using a total of 60 embryos. In a hatching assay conducted in tandem 100% of control embryos hatched, 92% between 56 and 72 hours, n = 12 (6 MO3 injected, 6 uninjected). In the same assay only 6% of MO injected embryos hatched, n = 18 (6 MO1, 6 MO2 and 6 MO4 injected). C: Substrate cleavage for single embryos. MO =  MO4 injected, Control =  MO3 injected. Data represents a typical release profile for a morphant and a control embryo.

The hatching gland releases enzymes that break down the chorion and allow embryos to hatch [38], [39], a process possibly involving *cd63*. *Cathepsin L* encodes a secreted gene product [40] and is a putative zebrafish hatching enzyme that is expressed at high levels in the hatching gland ([37] and figure 6B). The release of hatching enzymes was measured using Z-Phe-Arg-7-amido-4-methylcoumarin as a substrate for *cat L,* although it is also cleaved by other proteolytic enzymes. This lack of absolute specificity was desirable for this assay due to the potential presence of multiple zebrafish hatching enzymes. Dechorinated morphant embryos were assayed alongside mismatch MO and uninjected controls. No significant difference was seen in substrate cleavage between WT and mismatch MO injected embryos confirming the suitability of mismatch MO3 as a negative control (Figure 8A). Injection of experimental MO resulted in strong inhibition of substrate cleavage (Figure 8B) and the majority of MO injected embryos in a matched hatching assay did not hatch (Figure 8B, legend). In contrast, mismatch MO injected and uninjected controls had peak substrate cleavage (Figure 8B) at a time which corresponded to hatching in control embryos (Figure 8B, legend). Analysis of data from individual embryos revealed that in control embryos substrate cleavage was the result of a one-off release event, rather than a gradual release over a sustained period (Figure 8C). Taken together these results demonstrate that zebrafish have a single release event of enzymes capable of substrate cleavage that corresponds with hatching. *cd63* knockdown inhibits substrate cleavage suggesting that *cd63* has a specific role in the hatching mechanism.

## Discussion

Like many zebrafish genes [41], maternal *cd63* transcript is present during the blastula stage of embryogenesis. Early zygotic *cd63* expression was observed in important structures during development of zebrafish and this could signify a role for *cd63* during early development. Expression was apparent in the prechordal plate, which is the origin of the anterior mesoderm and gives rise to head and eye muscles as well as pharyngeal endoderm [28]. The polster is part of the prechordal plate and is the rudiment to the hatching gland. Given the widespread expression of CD63 orthologs it is surprising that in zebrafish expression is restricted in the fashion observed. It may be the case that *cd63* is expressed at low levels in tissues throughout the zebrafish, as would be expected by analogy with mammalian CD63, and that the high through put *cd63* ISH data published electronically [27], [33], as well as the ISH presented here, is too insensitive to detect low levels of expression. This possibility is supported by micro array data showing that *cd63* is over expressed in the zebrafish retinal pigment epithelium (RPE) relative to cells of the retina [42]. *cd63*, like other markers of anterior mesoderm, was not expressed in *oep* mutants. Further to this we observed that *gsc* and *catl* expression, as well as apparent tissue density in morphant embryos, was unaffected by *cd63* knockdown. From this we conclude that *cd63* is a nodally regulated gene and that expression is not required for the specification of hatching gland tissue.

MO-induced defects in both hatching gland morphology and hatching demonstrate a potential role for *cd63* in the organisation of hatching gland cells and the function of the secretory machinery. The phenotype seen in the hatching gland as a result of CD63 knock down is not unique to Cd63, as knockdown of *xbp1*, a protein reported to be involved in terminal differentiation of the secretory machinery of the hatching gland, resulted in reduced granule size and a similar morphology to that seen in this work. A further consequence of *xbp1* knock down was failure to hatch [43]. This raises the possibility that *xbp1* and *cd63* may have overlapping functions or be involved in the same hatching gland processes, as is also the case for *zKLF4*. Failure to hatch as a consequence of MO mediated knockdown of *zKLF4* was due to an extensive disruption to hatching gland morphology [44]. Our detailed microscopic analysis revealed that organisation of the gland was also disrupted by *cd63* knockdown, evident by mislocalisation of cells and an altered shape. This mislocalisation of cells may have been due to altered CD63-dependent integrin function, thereby affecting processes of migration and cellular adhesion to the extracellular matrix [45]. An alternative is that hatching enzymes are not delivered to granules properly in morphants, causing toxicity, or that the enzymes themselves are essential for correct migration.

The intracellular granules within the hatching gland cells were also seen to change from being bulbous and well organised in clusters, to small, misshapen and loosely clustered. Morphant granules rapidly moved in a seemingly random fashion, whereas movement in control cells was less obvious, slower and restricted to the immediate area local to the granule. The reason for these changes in granule movement is unknown but a similar effect occurs in mast cells from rab27b deficient mice [46], suggested to be due to a defect in the peripheral actin-mediated stationing of granules adjacent to the plasma membrane. Alternatively, this may be a consequence of reduced granule size allowing more room for movement in the actin network. In Hermansky-Pudlak syndrome, mutation of the β subunit of AP-3 results in mislocalisation of CD63, increased lysosome granule size and a failed release of lytic factors due to defective migration of intracellular granules [47]. In cytotoxic T lymphocytes from a Hermansky-Pudlak sufferer, lytic granules were unable to move along microtubules and dock with relevant secretory domains [47]. Further to this, siRNA mediated knockout of CD63 in a neutrophil model lead to depletion of granules [16], due to a failure in CD63 mediated trafficking of factors to populate the granules, which was accompanied by a defective granule morphology similar to that seen in the hatching gland cells of morphant embryos. Finally, in RBL-2H3 cells, CD63 specific antibodies block full degranulation of lysosomes [18]. It is possible the CD63-mediated mechanism of granule population with enzymatic cargo is conserved in the zebrafish hatching gland and therefore the defects seen as a result of *cd63* knockdown are similar. It seems likely that *cd63* is important for transport, and could be involved in transportation of factors both to and from the intracellular stores, which in turn may involve interaction with microtubules. A final possibility is that *cd63* may be required for correct production of enzymes, a role previously unassociated with CD63.

The phenotype of the CD63 knockout mouse (in the C57BL/6J background) was surprisingly subtle, with mild kidney and colon water retention defects [48]. Litter sizes were not reported to be smaller than expected, suggesting that blastocyst hatching, a process known to involve proteolytic enzymes, was not affected to any great extent. It is not clear if mammals have alternative mechanisms for the secretion of these enzymes or if other tetraspanins have been selected to compensate for CD63 deficiency during the knockout procedure. The mechanism of the water retention defects is also currently unclear.

In summary, *cd63* morphants have multiple hatching gland defects manifested as incorrect localisation, abnormal intracellular granules and excessive granule movement. *cd63* is a Nodally-regulated gene involved in hatching gland differentiation rather than specification. Disrupted hatching gland is accompanied by hatching defects. Zebrafish hatching is mediated by an enzyme release event, absent in morphants. The consequence of *cd63* knockdown is likely mediated through partner proteins of which there are many potential candidates for further investigation.

## Materials and Methods

### Ethics Statement

All experimentation in the CDBG conforms to the UK and EU guidelines on research practice (http://cdbg.shef.ac.uk/about/policy/index.html).

No specific ethics approval under UK and EU guidelines was required for this project, as all zebrafish used in this study were between 0 and 5 days old.

### Fish

All experiments used 0–5 day old London wild type embryos (maintained by the MRC Centre for Developmental and Biomedical Genetics, University of Sheffield) unless otherwise stated, maintained in E3 buffer (0.5 mM NaCl, 0.017 mM KCl, 0.033 mM CaCl_2_, 0.033 mM MgSO_4,_ 10^–6^% methylene blue). Morpholinos, plasmid DNA and RNA were injected into the yolk sac of embryos at the 1 cell stage.

### Transfection of mammalian cells

Cd63GFP was generated by insertion of the IMAGE *cd63* cDNA into the EGFP-N1 plasmid (Clonetch). Primers were designed to add a Sac II restriction site before the ATG of *cd63* and to mutate the G to a C in the stop codon, add two extra bases and an Age I restriction site. Sequential restriction digest of insert and vector was followed by clean up and ligation using T4 DNA Ligase (Promega). The Cd63GFP construct was transfected into mammalian cells by electroporation using the Gene Pulser II (Bio-Rad) or injected into the yolk of embryos. CHO or RBL-2H3 cells stably expressing Cd63GFP were selected by growth in DMEM-10% FCS containing G418 (Invitrogen), followed by florescence activated cell sorting using a FACS Aria (Beckman and Dickinson).

### Protein extraction and analysis

Lysates from mammalian cells or embryos were prepared by resuspension in 50 mM Tris (pH 8.0), 150 mM NaCl, 0.02% NaN_3_ with 1% CHAPS with 10% protease inhibitors (Sigma). After an incubation on ice for 30 minutes lysates were centrifuged at 13000 g for 30 minutes at 4°C to pellet nuclei. SDS-PAGE and Western blotting were carried out using standard procedures using and Bio-rad mini Protean and mini transblot transfer cell apparatus. Anti GFP antibody was supplied by Biosera.

### Microscopy and image analysis

Embryos were incubated in E3+4.2% tricaine before dechorination and mounting in E3-containing 3% methylcellulose. Imaging of cells by fluorescence microscopy used a Nikon Eclipse E400, and DIC microscopy (Axioscope, Zeiss; Jena, Jenoptik) was used for all other images and time lapse. Images were viewed using Adobe Photoshop CS2 and analysed using ImageJ (http://rsb.info.nih.gov.ij/). The brightness and contrast of some images were adjusted for clarity. All statistical analysis was performed using GraphPad Prism version 5.00 for Windows, GraphPad Software, USA, www.graphpad.com.

### Whole mount in situ hybridisation

IMAGE clones (Geneservice Ltd.) of *cd63* (BC056698), *cd82* (BC054586) and *catl* (BC108031) were used as template to generate digoxigenin labelled in situ RNA probes using DIG RNA labelling mix (Roche). Whole mount in situ hybridisation was performed as described previously [49] with the following modifications: hybridisation was conducted at 65°C, PBT (1X PBS 0.1% Tween-20), 2% FCS (foetal calf serum) was used to block non-specific antibody binding and anti digoxigenin Fab fragments (Roche) were used to detect bound probe with detection achieved using NBT/BCIP (Sigma).

### Antisense morpholino oligonucleotide and RNA injection

Four morpholino oligonucleotides (MO, Gene Tools) were designed to target the 5′ CD63 sequence. MO1 (5′-TTTCGCTCCTCCTTCTACAGCCATG-3′) was designed against the start codon to block translation; MO2 (5′-ACAGCACTTGAGCTAATGGAAAAAC-3′) and MO4 (5′-TGCAAACTGAGGGAACACAAAGAT-3′) were targeted against the splice donor site of exon 6 and exon 4 respectively to disrupt splicing and MO3 (5′-TTTCCCTGCTGCTTATACAGCGATG-3′) was a 5 base mispair control for MO1. Initially 3–8 pg MO1 and 4–12 pg of MO2 or MO4 were injected into single cell embryos to determine an optimal experimental dose. In further experiments MO2 and MO4 were used at a dose of 10 pg and MO1 at a dose of 1 pg in order to balance specific phenotype (Figure 4B and C; 5A II) against typical morpholino-induced non-specific defects (such as oedema and a crooked body plan). The data presented in this work is from MO4 injected embryos for which we obtained direct molecular evidence for its action (Figure 3B), but similar results were seen with MO1 and 2.

### RT-PCR

Total RNA was extracted from single embryos homogenised in Tri Reagent (Ambion) following the manufacturer's instructions and cDNA generated from subsequent extracts using SuperScript II reverse transcriptase (Invitrogen) and oligo dT (Invitrogen) following the manufacturer's instructions. PCR was conducted on resulting cDNA using primers 5′-GTTTCGCTTTGCTGA-3′ and 5′-AAAGAGATGAAGAAGATGACGC-3′ to generate full length *cd63* as well as with primers 5′-GCCCCTGCCAATGTAACCAC-3′ and 5′-TGCCAGGGACCATCTCAACAA-3′ to generate a fragment of elongation factor EF1a as a control. PCR conditions were 95°C 5 minutes, 30x 95°C 30 seconds, 30x 55°C 30 seconds, 30x 72°C 2 minutes, 72°C 5 minutes. Resulting PCR products were electrophoresed on 1.2% agarose gels containing 0.5 µgml ethidium bromide.

### Hatching assays

Embryos were incubated in petri dishes in E3 at 28.5°C. Media was changed once a day with minimal disturbance to embryos. A hatching event was recorded when an embryo became free of its chorion.

### Cathepsin L assay

The cathepsin L assay was developed through adaptation of an established Calbiochem protocol supplied by Merck Chemicals Ltd, based on the capacity of z-Phe-Arg-7-amido-4-methylcourmarin to be cleaved by cathepsin L to release the fluorescent product 7-amido-4-methylcoumarin (AMC). AMC was quantified using a spectrophotometer (WPA Lightwave; excitation of 370 nm, emission of 460 nm). The assay was carried out by placing single dechorinated embryos into wells of a v-bottomed 96 well plate in 110 µl E3 containing 5 mM HEPES. The plates were sealed and incubated at 28°C. After 4 hours a 100 µl sample of E3 was removed from each well and immediately replaced with 100 µl of preheated fresh E3. The plate was then returned to 28°C. 50 µl of assay buffer (340 mM sodium acetate, 60 mM acetic acid, 4 mM EDTA P.H 5.5, 8 mM DTT) was added to a 100 µl sample, and incubated at 30°C for 1 minute. 50 µl of 20 µM substrate was then added and incubated at 30°C for a further 10 minutes. The reaction was stopped by addition of 200 µl of stop solution (100 mM sodium monochloroacetate, 70 mM acetic acid, 30 mM sodium acetate). This process was repeated on a four hourly basis for the duration of the assay. A hatching control, consisting of individual embryos with intact chorions was carried out in spare wells of the assay plate. Control wells were treated identically to experimental wells and therefore were exposed to the same temperature fluctuations as dechorionated embryos. In these matched control embryos, hatching from the chorion was also recorded.

## Supporting Information

Figure S1 Schematic representation of zebrafish Cd63 protein. Residue colour changes indicate separate exons within the reading frame of cd63. Solid black lines between residues denote disulphide bonds.
(TIF)

Figure S2 A. Localisation of Cd63GFP in 24 hr LWT embryos injected with buffer or plasmid DNA encoding Cd63GFP or EGFP, as indicated. Scale bars  = 52 µm. B. Zoom of area of fluorescent Cd63GFP and GFP images in S2 A. Scale bars  = 52 µm.
(TIF)

Movie S1 Typical WT Hatching Gland. The time lapse is one frame every 5 seconds for a total of 3 minutes (36 frames), using a 40x objective. Little movement of intracellular hatching gland granules is apparent.
(MOV)

Movie S2 Typical morphant Hatching Gland. The time lapse is one frame every 5 seconds for a total of 3 minutes (36 frames), using a 40x objective. The morphology of the intracellular granules in morphant embryos is altered and there is a large amount of granule movement when compared to WT.
(MOV)
