# Supplementary figures and images for: Regulation of Zebrafish Hatching by Tetraspanin cd63
Source: PLoS One. 2011 May 19;6(5):e19683. doi: 10.1371/journal.pone.0019683 (PMC3098263; doi:10.1371/journal.pone.0019683)

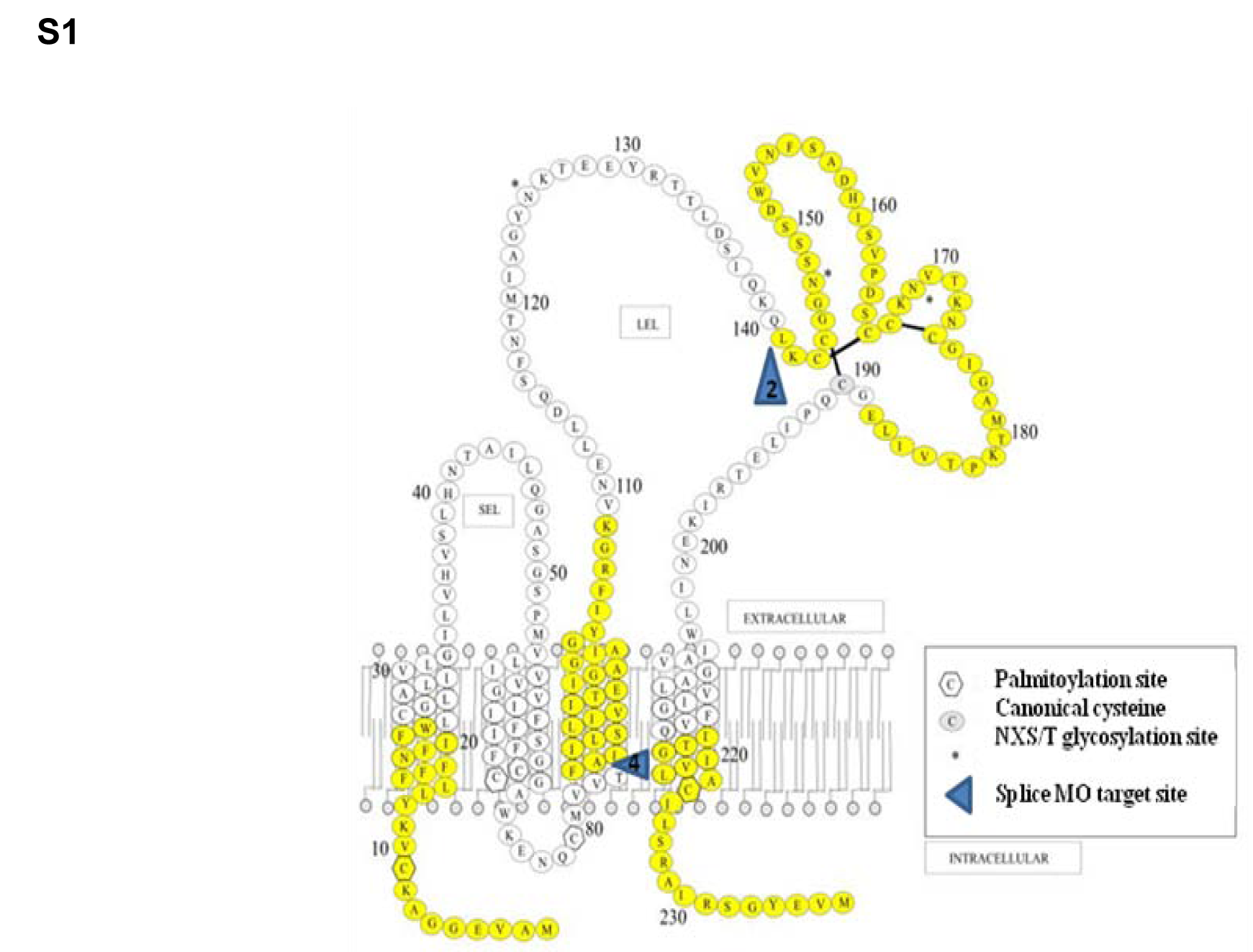

Supplement: Figure S1 — Schematic representation of zebrafish Cd63 protein. Residue colour changes indicate separate exons within the reading frame of cd63. Solid black lines between residues denote disulphide bonds. (TIF) [file pone.0019683.s001.tif]

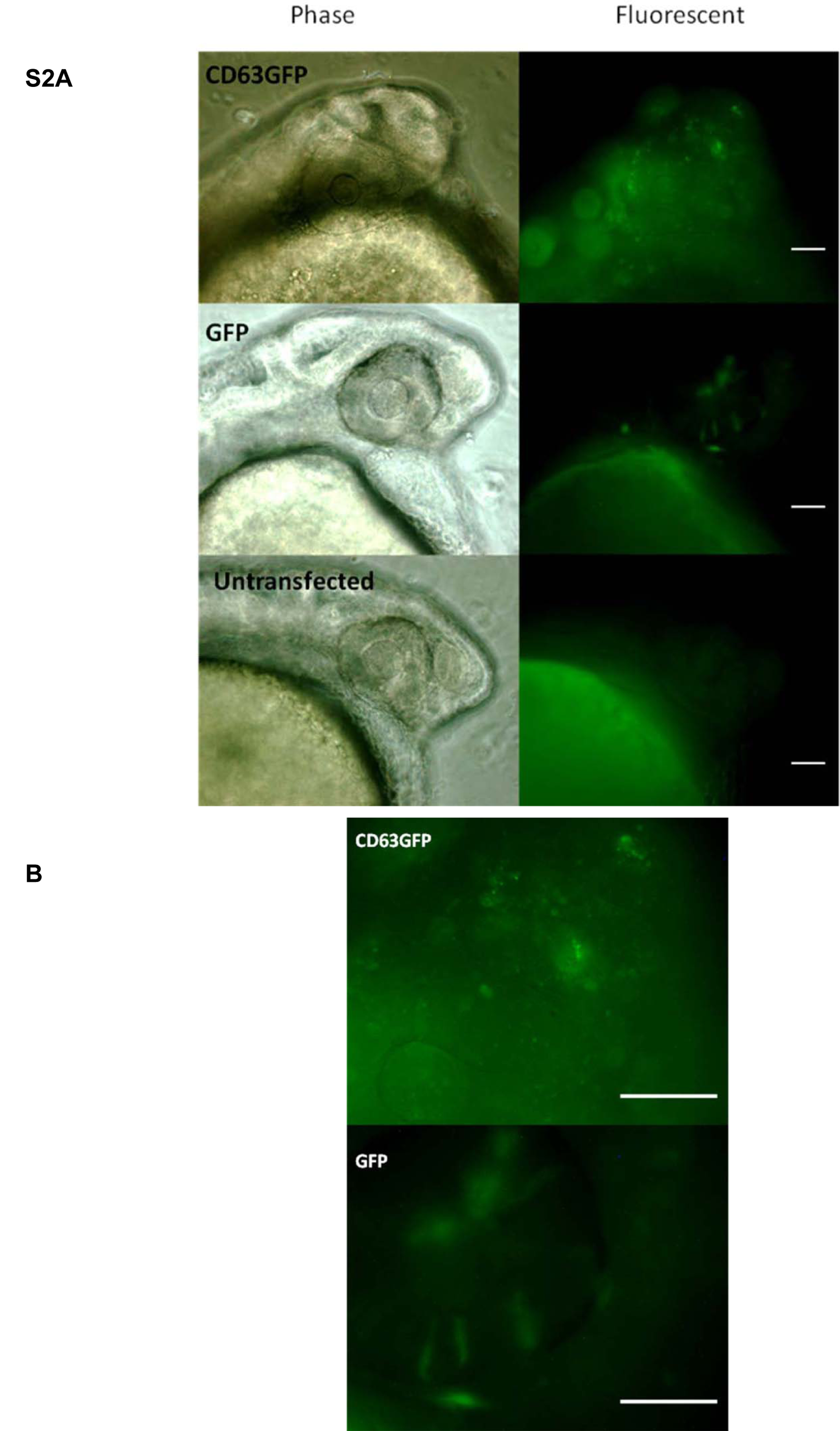

Supplement: Figure S2 — A. Localisation of Cd63GFP in 24 hr LWT embryos injected with buffer or plasmid DNA encoding Cd63GFP or EGFP, as indicated. Scale bars = 52 µm. B. Zoom of area of fluorescent Cd63GFP and GFP images in S2 A. Scale bars = 52 µm. (TIF) [file pone.0019683.s002.tif]
